# Acute severe mitral regurgitation: consideration of papillary muscle architecture

**DOI:** 10.1186/1476-7120-6-5

**Published:** 2008-01-18

**Authors:** Andrew Czarnecki, Amar Thakrar, Tielan Fang, Matthew Lytwyn, Roien Ahmadie, Edward Pascoe, Davinder S Jassal

**Affiliations:** 1 Department of Internal Medicine, St. Boniface General Hospital, Winnipeg, Manitoba, Canada; 2 Department of Internal Medicine, Royal University Hospital, Saskatoon, Saskatchewan, Canada; 3 Institute of Cardiovascular Sciences, St. Boniface Research Centre, Winnipeg, Manitoba, Canada; 4 Cardiac Surgery Division, Department of Cardiac Sciences, St. Boniface General Hospital, Winnipeg, Manitoba, Canada; 5 Cardiology Division, Department of Cardiac Sciences, St. Boniface General Hospital, Winnipeg, Manitoba, Canada; 6 Department of Radiology, St. Boniface General Hospital, Winnipeg, Manitoba, Canada

## Abstract

We present a case of an individual who presented with acute severe mitral regurgitation in the setting of an inferior ST elevation myocardial infarction. Both transthoracic and transesophageal echocardiography demonstrated a posteriorly directed eccentric jet of severe mitral regurgitation with flail anterior mitral valve leaflet attached presumably to the anterior papillary muscle. Intraoperative findings demonstrated rupture of the postero-medial papillary muscle attached via chords to the anterior mitral valve leaflet. This case serves to remind us that both the anterior and posterior leaflets of the mitral valve are attached to both papillary muscle heads. The direction and eccentricity of the mitral regurgitant jet on echocardiography helps to locate the leaflet involved, but not necessarily the coexisting papillary muscle pathology.

## Introduction

Echocardiography is the primary imaging modality of choice for the noninvasive assessment of mechanical complications such as acute mitral regurgitation in the setting of myocardial infarction (MI). Echocardiographic features of importance include location of the papillary muscle rupture and leaflet involvement, direction and severity of mitral regurgitation, and hemodynamic complications. We report a case of an individual in whom both transthoracic and transesophageal echocardiography demonstrated a posteriorly directed eccentric jet of severe mitral regurgitation due to a flail anterior mitral valve leaflet with presumed anterior papillary muscle rupture. Intraoperative findings confirmed rupture of the postero-medial papillary muscle attached via chords to the flail anterior mitral valve leaflet.

## Case presentation

A 62 year-old female presented to hospital with a two day history of retrosternal chest discomfort, shortness of breath, nausea and diaphoresis. The patient was tachycardic, hypotensive, with a room air oxygen saturation of 83%. Jugular venous pressure was elevated at the angle of the jaw with prominent v wave. Initial cardiorespiratory examination was remarkable for an S3 with bibasilar crackles.

The complete blood count, electrolytes and liver function tests were within normal limits. The cardiac enzymes including troponon I and creatine kinase were elevated consistent with myocardial injury. The baseline electrocardiogram was consistent with an inferior ST-elevation myocardial infarction. Chest x-ray demonstrated pulmonary vascular redistribution.

**Figure 1:**
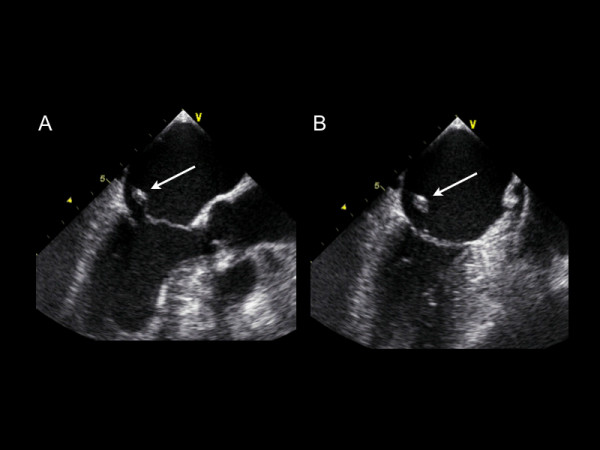
**A-B **A transesophageal echocardiographic long axis and two chamber midesophageal view illustrating a flail anterior mitral valve leaflet with ruptured papillary muscle.

**Figure 2:**
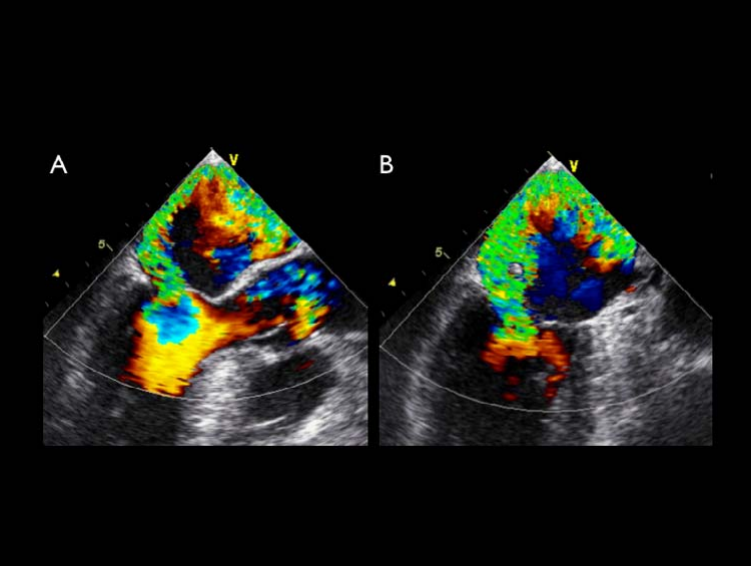
**A-B **A transesophageal echocardiographic long axis and two chamber midesophageal view with color Doppler demonstrating an eccentric posteriorly directed jet of severe mitral regurgitation.

After failed thrombolytics, the patient's cardiac catheterization revealed a distally occluded right coronary artery, not amenable to percutaneous coronary intervention. An intra-aortic balloon pump was inserted and she was transferred to the intensive care unit where urgent transthoracic echocardiography (TTE) revealed an eccentric, posteriorly directed jet of severe mitral regurgitation with a presumed rupture of the antero-lateral papillary muscle attached to the flail anterior mitral valve leaflet. This finding was confirmed by transesophageal echocardiography (TEE) (Figures 1 and 2) [See Additional files 1, 2, 3, 4]. Transgastric views using TEE were suboptimal.

During cardiopulmonary bypass, one head of the posterior papillary muscle was ruptured and attached via chordae to the flail anterior mitral leaflet. A mechanical mitral valve was implanted and single vessel coronary artery bypass was performed. The patient was discharged from hospital on the fifth post-operative day.

## Discussion

Acute mitral regurgitation is a life threatening complication of myocardial infarction. In the setting of an inferior MI, a high index of suspicion for this complication should be entertained. In an era of competing noninvasive cardiac imaging modalities including echocardiography, computed tomography, and magnetic resonance imaging, TTE has been well established as the initial diagnostic tool used to identify rupture of a papillary muscle with a diagnostic sensitivity of 65–85% [1-5]. Due to the close proximity of the ultrasound transducer to the mitral apparatus, TEE is often used to better delineate the cause of MR and improves the diagnostic yield to between 95–100%. Colombo et al. demonstrated, however, that the sensitivity of both TTE and TEE for papillary muscle rupture improves significantly with measurement of eccentric jets of mitral regurgitation (MR) using color Doppler [6]. By calculating the angle of the proximal MR jet and the plane of the mitral annulus, an angle of ≤ 47° on TEE and ≤ 45° on TTE established a sensitivity and specificity of 88% for flail mitral leaflet [6].

The mitral valve is located retrosternally at the fourth costal cartilage, consisting of an anterior and posterior leaflet, chordae tendinae, papillary muscles, ventricular wall and annulus connected to the atria. Each leaflet is supported by chordae tendinae that are attached to papillary muscles which become taut with each ventricular contraction preserving valvular competence. Both the anterior and posterior leaflets of the valve are attached via primary, secondary and tertiary chordae to both the antero-lateral and posterio-medial papillary muscles. A disruption in either papillary muscle in the setting of myocardial injury, can result in dysfunction of either the anterior or posterior leaflet of the mitral valve.

Our case illustrates a posteriorly directed jet of severe MR caused by a flail anterior leaflet of the mitral valve with rupture of the poster-medial papillary muscle. In the setting of an inferior STEMI, disruption of flow in the right coronary artery often leads to posterior papillary muscle rupture due its single blood supply. Although TTE and TEE demonstrated complete disruption of the anterior mitral leaflet, direction of an eccentric jet does not always correctly predict the site of papillary muscle rupture. Complete visualization of papillary muscle involvement through a number of imaging planes is required. It is important to remember the architecture of the papillary muscles and mitral valve leaflets in the noninvasive assessment of MR by echocardiography.

## Supplementary Material

Additional file 1 Movie 1A. A transesophageal echocardiographic long axis midesophageal view illustrating a flail anterior mitral valve leaflet with ruptured papillary muscle. Click here for file

Additional file 2 Movie 1B. A transesophageal echocardiographic 2-chamber midesophageal view illustrating a flail anterior mitral valve leaflet with ruptured papillary muscle. Click here for file

Additional file 3 Movie 2A. A transesophageal echocardiographic long axis midesophageal view with color Doppler demonstrating an eccentric posteriorly directed jet of severe mitral regurgitation. Click here for file

Additional file 4 Movie 2B. A transesophageal echocardiographic two chamber midesophageal view with color Doppler demonstrating an eccentric posteriorly directed jet of severe mitral regurgitation. Click here for file
